# Induced defense response in red mango fruit against *Colletotrichum gloeosporioides*

**DOI:** 10.1038/s41438-020-00452-4

**Published:** 2021-01-10

**Authors:** Pradeep Kumar Sudheeran, Noa Sela, Mira Carmeli-Weissberg, Rinat Ovadia, Sayantan Panda, Oleg Feygenberg, Dalia Maurer, Michal Oren-Shamir, Asaph Aharoni, Noam Alkan

**Affiliations:** 1 Department of Postharvest Science of Fresh Produce, Agricultural Research Organization, Volcani Center, P.O. Box 15159, HaMaccabim Road 68, Rishon LeZion, 7505101 Israel; 2 Department of Plant Pathology and Weed Research, ARO, Volcani Center, Rishon LeZion, 7505101 Israel; 3 Department of Fruit Tree Sciences, Agricultural Research Organization, Volcani Center, PO Box 6, Bet-Dagan, 7505101 Israel; 4 Department of Ornamental Plants and Agricultural Biotechnology, ARO, Volcani Center, P.O. Box 15159, HaMaccabim Road 68, Rishon LeZion, 7505101 Israel; 5 Department of Plant and Environmental Sciences, Weizmann Institute of Science, Rehovot, 7610001 Israel

**Keywords:** Biotic, Secondary metabolism

## Abstract

Mango fruit exposed to sunlight develops red skin and are more resistant to biotic and abiotic stresses. Here we show that harvested red mango fruit that was exposed to sunlight at the orchard is more resistant than green fruit to *Colletotrichum gloeosporioides*. LCMS analysis showed high amounts of antifungal compounds, as glycosylated flavonols, glycosylated anthocyanins, and mangiferin in red vs. green mango skin, correlated with higher antioxidant and lower ROS. However, also the green side of red mango fruit that has low levels of flavonoids was resistant, indicated induced resistance. Transcriptomes of red and green fruit inoculated on their red and green sides with *C. gloeosporioides* were analyzed. Overall, in red fruit skin, 2,187 genes were upregulated in response to *C. gloeosporioides*. On the green side of red mango, upregulation of 22 transcription factors and 33 signaling-related transcripts indicated induced resistance. The RNA-Seq analysis suggests that resistance of the whole red fruit involved upregulation of ethylene, brassinosteroid, and phenylpropanoid pathways. To conclude, red fruit resistance to fungal pathogen was related to both flavonoid toxicity and primed resistance of fruit that was exposed to light at the orchard.

## Introduction

Numerous studies have shown that flavonoids and anthocyanins play a significant role in plant resistance to both biotic and abiotic stresses; as such, they can be divided into constitutive and induced compounds^1^. Biotic and abiotic stresses induce resistance in plants, priming the defense mechanism to protect the plant against future challenges^2,3^. This primed defense response often includes induction of the phenylpropanoid-biosynthesis pathway, which results in the accumulation of flavonoids and anthocyanin pigments in response to low temperature, intense light exposure, wounding, or pathogen infection^1,4^. The plant response to pathogens is governed by salicylic acid, jasmonic acid, ethylene, and other phytohormones, which activate signaling pathways and plant defense responses, including the phenylpropanoid pathway^5,6^.

Mango fruit (cvs. Shelly, Kent, and Maya) exposed to direct sunlight in the orchard accumulate flavonols and anthocyanins^4,7^. These red mango fruit is more resistant to postharvest fungal pathogens and chilling injury^7,8^.

Flavonols also play a part in the constitutive defense response^1^. In a recent publication, we found a high accumulation of flavonols and anthocyanins, mostly in their glycosylated form, in the skin of red mango fruit; these compounds exhibited direct antifungal activity against fungal pathogens such as *Colletotrichum*^9^. Interestingly, as the fungi attack, it secretes *β*-glucosidase, which results in aglycon flavonoids that were more toxic to the fungi^9^.

Anthracnose, caused by the fungus *Colletotrichum gloeosporioides*, is a destructive disease in over 470 plant species^10^, and is the most destructive disease in mango fruit (*Mangifera indica* L.)^11^. The interaction between *C. gloeosporioides* and tomato fruit has been studied previously^12^. Moreover, the transcriptome of harvested mango cv. Zill fruit responding naturally to *C. gloeosporioides* infection was characterized^13^.

To better understand the resistance of red mango fruit to *C. gloeosporioides*, the present study profiled the red and green fruit transcriptomes and phenylpropanoid metabolites in response to fungal infection. The main finding of this work was that the red fruit that was exposed to sunlight at the orchard both accumulates flavonoids on the red side of the fruit and activates an antifungal defense response that includes jasmonic acid, ethylene, and phenylpropanoid biosynthesis. This information on red fruit tolerance could provide an excellent platform for further understanding the interactions between mango fruit and pathogens, with the aim of developing future means for controlling mango disease.

## Results

### Evaluation of mango fruit color and physico-chemical properties

‘Shelly’ mango fruit has red and green skin. Fruit grown on the exterior of the canopy has a prominent red skin color and is referred to in this study as RF (Supplementary Fig. S1A). Fruit growing in the shaded interior of the tree canopy, has mostly green skin, and is referred to as GF (Supplementary Fig. S1A). Quantitatively, RF had over 60% of their skin-colored red, and GF had less than 10% of their skin-colored red. Measurements of harvested fruit at the greenest point showed a hue of 120 on the green sides (correlating with green color) of both RF and GF, while a hue of 29 (correlating with red color) and 118 were found on the reddest point of the red sides of RF and GF, respectively (Supplementary Fig. S1B).

The fruit was also evaluated for physiological quality using the parameters firmness, Brix, and acidity, measured at harvest. Both RF and GF showed similar firmness at harvest (Supplementary Fig. S1C). However, RF acidity content was non-significantly lower (Supplementary Fig. S1E), and its TSS content was slightly higher (Supplementary Fig. S1D) than in the GF. Therefore, GF and RF had relatively similar ripening parameters.

### LC–MS analysis

**Fig. 1:**
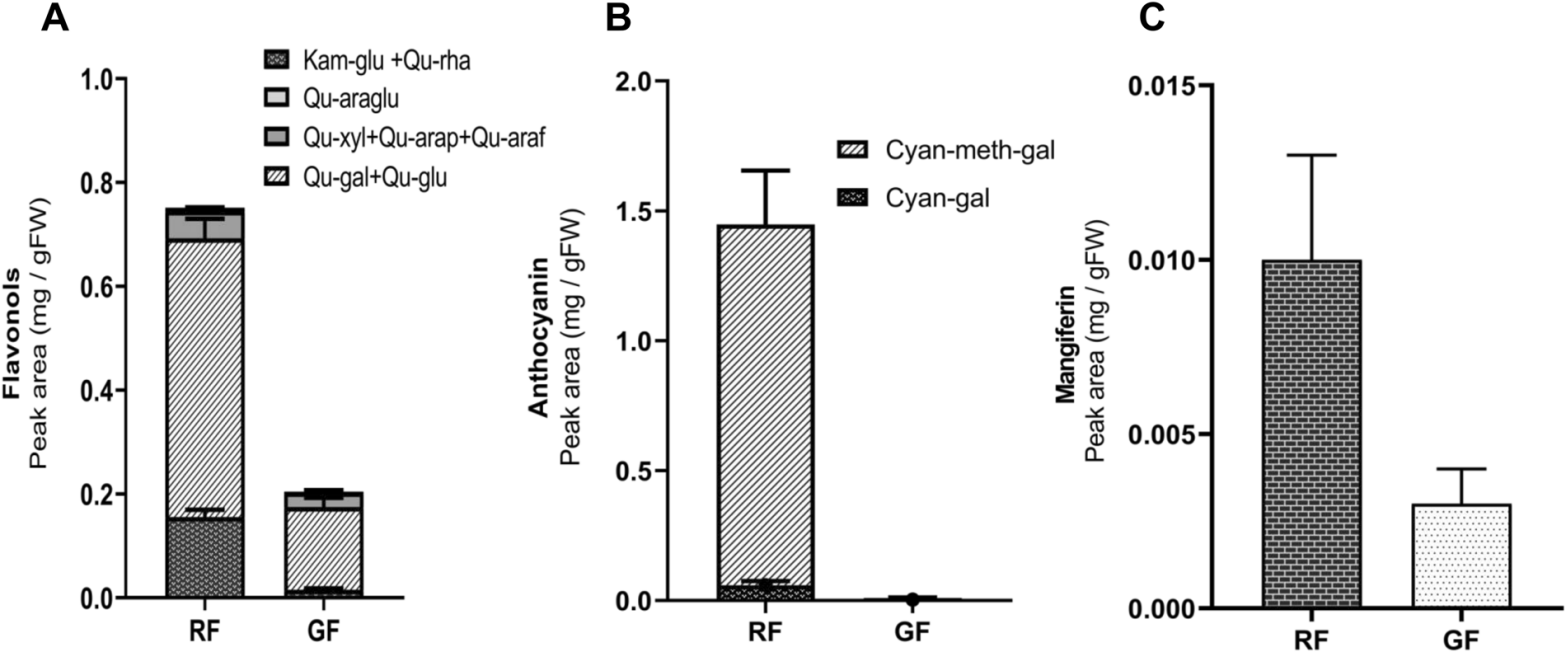
Polyphenol contents in red and green fruit. Quantification of flavonols **A**, anthocyanins **B**, and mangiferin **C** in the skin of red and green mango fruit (RF and GF, respectively) at harvest by LC–MS, represented by peak area. Flavonols (Qu-araglu, quercetin 3-O-arabinoglucoside; Qu-xlu, quercetin 3-O-xyloside; Qu-arap, quercetin 3-O-arabinopyranoside;Qu-araf, quercetin 3-O-arabinofuranoside; Qu-gal, quercetin 3-O-galactoside; Qu-glu,quercetin 3-O-glucoside; Qu-rha, quercetin 3-O-rhamnoside; Kam-glu, kaempferol 3-O-glucoside) and anthocyanin (Cyan-gal,cyanidin 3-O-galactoside; Cyan-meth-gal, 7-O-methylcyanidin)

Anthocyanin and flavonol compounds were characterized in the skin of RF and GF at harvest using UPLC–QTOF–MS/MS. The highest amount of glycosylated flavonols was detected in the RF skin, whereas the GF skins had a low amount of these metabolites (Fig. 1a). The major flavonol metabolites were mostly quercetin and kaempferol glucoside derivatives: quercetin 3-O-galactoside (retention time 2.45 min), quercetin 3-O-glucoside (2.49 min), quercetin 3-O-xyloside (2.59 min), quercetin 3-O-arabinopyranoside (2.65 min), quercetin 3-O-arabinofuranoside (2.68 min), quercetin 3-O-rhamnoside (2.80 min), quercetin 3-O-arabinoglucoside (2.24 min), and kaempferol 3-O-glucoside (2.75 min).

The amount of anthocyanins was also significantly higher in the skin of RF compared to GF (Fig. 1b). Two major anthocyanins were characterized, with dominance of 7-O-methylcyanidin (2.46 min) and a minor amount of cyanidin 3-O-galactoside (1.52 min). LC–MS analysis also showed a higher amount of the bioactive compound mangiferin in RF vs. GF (Fig. 1c).

### Red mango fruit resistance to pathogen

**Fig. 2:**
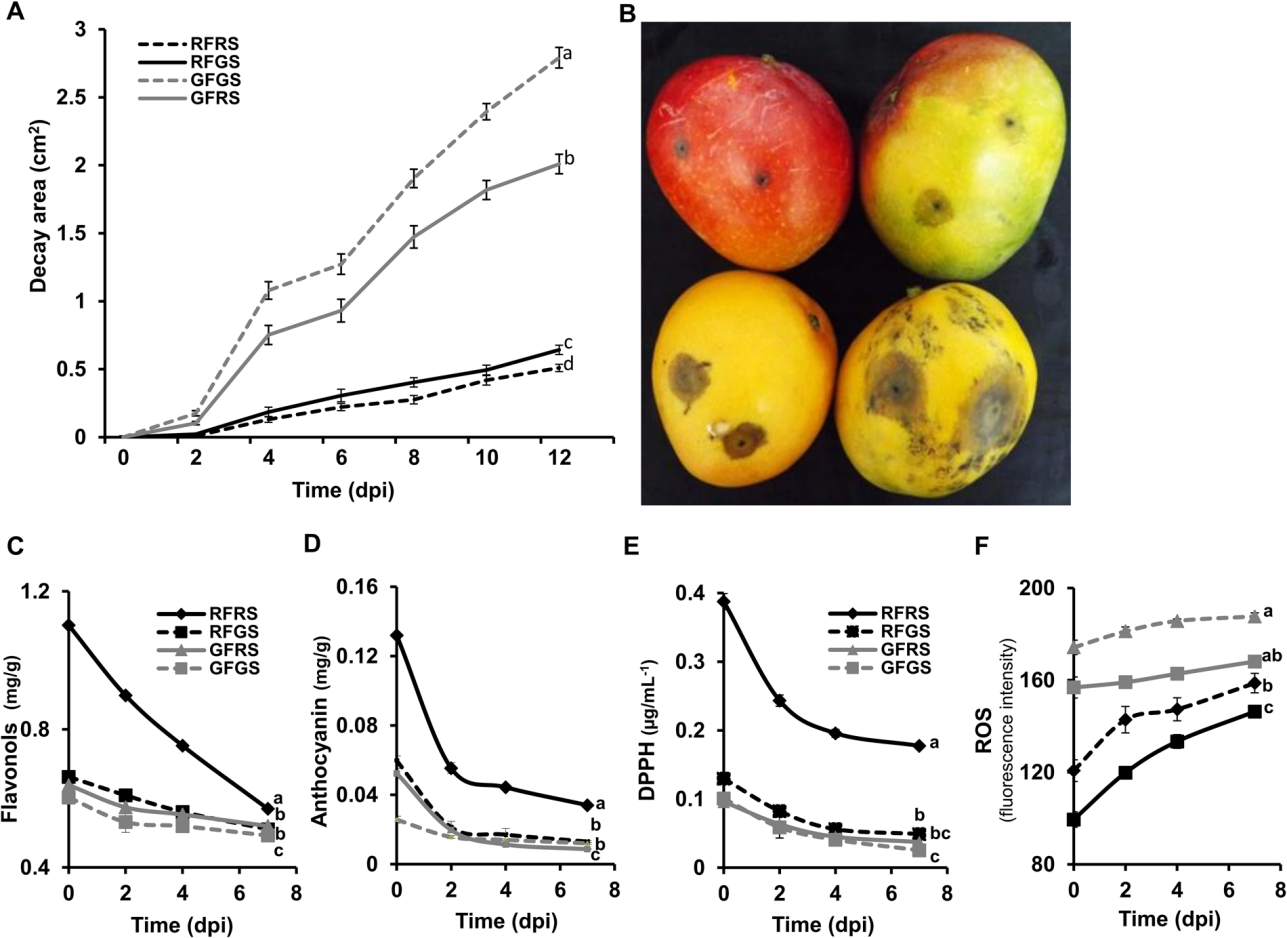
Resistance to *C. gloeosporioides* and quantification of anthocyanins, flavonols, antioxidant activity, and ROS production in red and green mango fruit (RF and GF, respectively). **A** Area of *C. gloeosporioides* decays on red and green sides (RS and GS, respectively) of RF and GF for 12 days post-inoculation (dpi). Values are means ± SE (*n* = 120). **B** Representative picture of (clockwise from top left): RFRS, RFGS, GFRS, GFGS 12 dpi with *C. gloeosporioides*. Total flavonoid (**C**) and anthocyanin (**D**) contents in mango skin. **E** Antioxidant activity of DPPH. **F** ROS production measured by the fluorescence intensity of DCF in the infection zones. Different letters indicate a significant difference (*p* < 0.05) according to Tukey–Kramer HSD test

Red and green mango fruits were inoculated with *C. gloeosporioides* and monitored for 12 days. On 2 to 12 dpi, the GF developed significantly (1.5- to 5.8-fold) larger decay areas than the RF, indicating that the latter were more resistant to necrotrophic colonization by *C. gloeosporioides* (Fig. 2a, b). Interestingly, both the red and green sides of the RF were more resistant to the fungi than the GF (Fig. 2a, b), indicating induced resistance on the green side of the RF.

### Assessment of flavonoid and anthocyanin content, ROS, and antioxidants in infected mango fruit

The total amount of flavonoids and anthocyanins was estimated chemically for infected RF and GF 0–7 dpi. The red side of the RF had a 2-fold higher level of flavonoids and anthocyanins compared to its green side and both sides of the GF (Fig. 2c, d). During fungal colonization and disease progress (0–7 dpi), the levels of both flavonoids and anthocyanins decreased significantly. However, at all evaluated time points, the flavonoid and anthocyanin levels of the red side of RF were significantly higher than those in the GF (Fig. 2c, d).

Next, antioxidant activities were measured in methanolic extracts of the infected zones of the fruit skin by monitoring the radical-scavenging activity of DPPH. Before inoculation, the red side of the RF had significantly higher activity (2-fold) than its green side and both sides of the GF (Fig. 2e). During fungal colonization and disease progress (0–7 dpi), antioxidant activity decreased in all samples, while the red side of the RF remained with the highest levels of antioxidant activity during all stages of disease progress (Fig. 2e).

Accumulation of ROS levels was detected by fluorescence microscopy using DCF staining of infected mango skin tissue from 0 to 7 dpi. The fluorescence intensity (relative ROS level) was significantly higher (2-fold) in the infected green side of the GF compared to the lower ROS levels detected in the RF (Fig. 2f). Similarly, the red side of the RF had less ROS than its green side, which had lower ROS levels than the GF. Relative ROS levels increased with time as the disease progressed, 0–7 dpi (Fig. 2f).

### Ethylene and respiration

Respiration rates and ethylene production were measured in the whole infected RF and GF for 10 days. Ethylene production in RF decreased from harvest to 10 dpi, whereas in GF, it remained high during the 10 dpi (Supplementary Fig. S2A). Respiration rates were measured by CO_2_ emission. Both RF and GF had increased respiration in response to the fungal infection. However, the RF showed a lower increase in respiration rate during infection (CO_2_ 0.289–1.084 percent) than the GF (CO_2_ 0.692–1.540 percent) (Supplementary Fig. S2B).

### Lipid peroxidation during infection

Spontaneous bioluminescent photon emission is positively correlated to lipid peroxidation^14,15^. During infection of RF and GF, 0–10 dpi, the luminescence of the whole fruit was measured with an IVIS. The infected and susceptible GF showed higher luminescence than the infected RF (Supplementary Fig. S3A and B). Similarly, the fluorescence intensity, which correlates with chlorophyll content, was higher in infected GF than RF (Supplementary Fig. S3C and D). Chlorophyll fluorescence decreased gradually during infection and fruit ripening in both RF and GF.

### Transcriptome analysis in RF and GF in response to *C. gloeosporioides* colonization

The transcriptomes of RF and GF inoculated with *C. gloeosporioides* on their red and green sides were analyzed at 0, 2, and 7dpi. The DEGs were screened with the criteria of *p* ≤ 0.01 (corrected for FDR) and 4-fold change in relative expression (log2 ≥ 2).

**Fig. 3:**
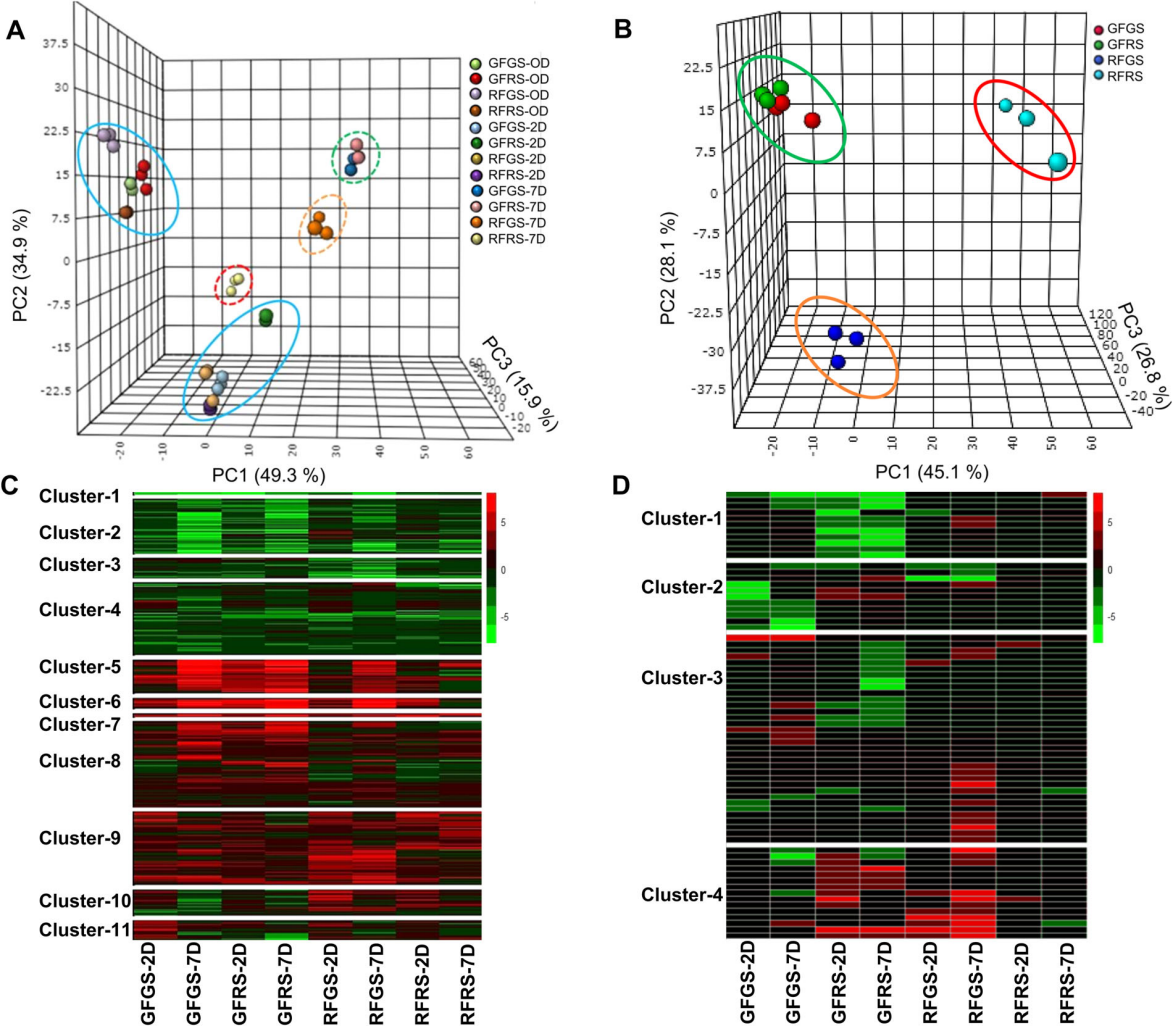
Simultaneous transcriptome analysis of mango fruit and *C. gloeosporioides*. Principal component analysis (PCA) and clustering heat map of mango fruit and *C. gloeosporioides* at different time points during the host-pathogen interaction. **A** PCA of red and green mango transcriptomes on 0, 2, and 7 days post-inoculation (dpi). Circles around the points represent different time points (0 dpi, 2 dpi), 7dpi-separated groups are circled in dashed line, **B** PCA of *C. gloeosporioides* transcriptome on 7D, separated groups are circled. **C** Heat map of differentially expressed genes in mango transcriptome at 2 dpi and 7 dpi vs. 0 dpi with 11 clusters. **D** Heat map of differentially expressed genes in *C. gloeosporioides* transcriptome with 4 clusters. RFRS, red fruit red side; RFGS; red fruit green side; GFRS, green fruit red side; GFGS, green fruit green side. *Z*-scores represent rescaled normalized log2 fold-change values for PCA and Heatmaps

The expression patterns of 2,187 DEGs at 2 dpi and 7 dpi vs. 0 dpi were subjected to hierarchical clustering, producing 11 clusters that were visualized on a heat map (Fig. 3c and Supplementary Fig. S4). Similarly, transcriptome analysis of the *C. gloeosporioides* used to inoculate the mango fruit showed 71 DEGs at 2 and 7 dpi that were clustered into 4 distinct clusters based on their expression patterns (Fig. 3d and Supplementary Table S1). The mango transcriptome of DEGs were analyzed by the 3D PCA plot. The samples could be divided into three distinct groups: harvest, 2 dpi, and 7 dpi (Fig. 3a). Interestingly, prior to fruit inoculation, at time 0, and initial colonization (2 dpi), the samples were grouped mainly based on time point, whereas, at 7 dpi, the transcriptomes of both sides of the GF were grouped, whereas those from the red side of the RF were closer to the samples from 2 dpi. The transcriptome of the green side of the RF was located between those of the red side of the RF and the GF (Fig. 3a).

A similar PCA for *C. gloeosporioides* colonizing mango fruit 7 dpi was studied (Fig. 3b). The fungal samples colonizing both sides of the GF clustered separately from those colonizing both sides of the RF (Fig. 3b).

**Fig. 4:**
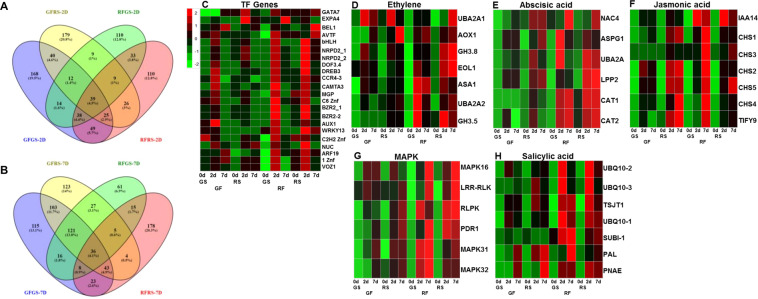
Induced defense response on the green side of red mango fruit (RFGS) in response to *C. gloeosporioides* colonization. **A**, **B** Venn diagrams representing common and distinct gene expression during 2 and 7 days post-inoculation through various comparisons. **C** Heat map of transcription factor genes induced in RFGS 2 dpi. **D**–**H** Heat maps of genes upregulated in RFGS after infection in the proposed pathways are related to signaling of ethylene, abscisic acid, jasmonic acid, MAP kinase (MAPK), and salicylic acid. *Z*-scores represent rescaled normalized log2 fold-change values. Abbreviations, transcript identification, and expression profile are presented in Table S2A, B. GS, green side; RS, red side; GF, green fruit; RF, red fruit; RFRS, red fruit red side; RFGS; red fruit green side; GFRS, green fruit red side; GFGS, green fruit green side

Venn diagrams of the DEGs at the different sampling time intervals (2 dpi and 7 dpi vs. time 0) showed that most DEGs were distinct for the different fruit colors. At 2 dpi, the green side of the GF showed 385 DEGs, its red side showed 339 DEGs, the green side of the RF showed 264 DEGs, and its red side showed 329 DEGs. At 7 dpi, the green side of the GF showed 465 DEGs, its red side showed 462 DEGs, the green side of the RF showed 289 DEGs, and its red side showed 312 DEGs (Fig. 4a, b).

The enriched GO terms that were upregulated significantly (4-fold) in the RF, compared to GF, in response to *C. gloeosporioides* inoculation (clusters 1, 2, 10, and 11) total of 178 analyzed genes. As expected, many GO terms were related to the plant’s response to biotic and abiotic stresses (Supplementary Fig. S5 and Supplementary Table S4).

The transcriptome was validated for key genes related to (heat shock protein [*HSP*], lipoxygenase [*LOX*]), and some of the signaling and phenylpropanoid pathway genes like polyubiquitin 10 (UBQ10_-_1), mitogen-activated protein kinase 16 (MAPK16), L-phenylalanine ammonia-lyase (PAL), Flavanone 3-hydroxylase (F3H_-_1), Flavanone 3-hydroxylase (F3H_-_2) by qRT-PCR. Linear regression analysis gave an R^2^ value of 0.824, indicating a close correlation between transcript abundance quantified by qRT-PCR and the transcript profile obtained from the RNA-Seq data, thereby supporting the latter’s accuracy (Supplementary Fig. S6).

### Identification of defense-related pathways in RF in response to *C. gloeosporioides* colonization

To look for genes that might be related to fruit resistance, we examined 110 upregulated DEGs in the samples from the green side of the RF (Fig. 4). Of these, 22 transcription factors were significantly induced at 2 dpi (Fig. 4a and Supplementary Table S2). Other relevant genes that were upregulated during infection at 2 or 7 dpi on the green side of the RF were related to phytohormones and the defense response and included transcripts related to ethylene (*ASA1, AOX1, EOL1, GH3.5, GH3.8, UBA2A1, UBA2A2*), Abscisic acid (*ASPG1, CAT1, CAT2, LPP2, NAC4, UBA2A*), Jasmonic acid (*CHS1, CHS2, CHS3, CHS4, CHS5, IAA14, TIFY9*), MAP kinase (*LRR-RLK, PDR1, RLPK, MAPK16, MAPK31, MAPK32*), and Salicylic acid (*PAL, PNAE, SUBI-1, TSJT1, UBQ10.1, UBQ10.2, UBQ10.3*) (Fig. 4d–h and Supplementary Table S2).

**Fig. 5:**
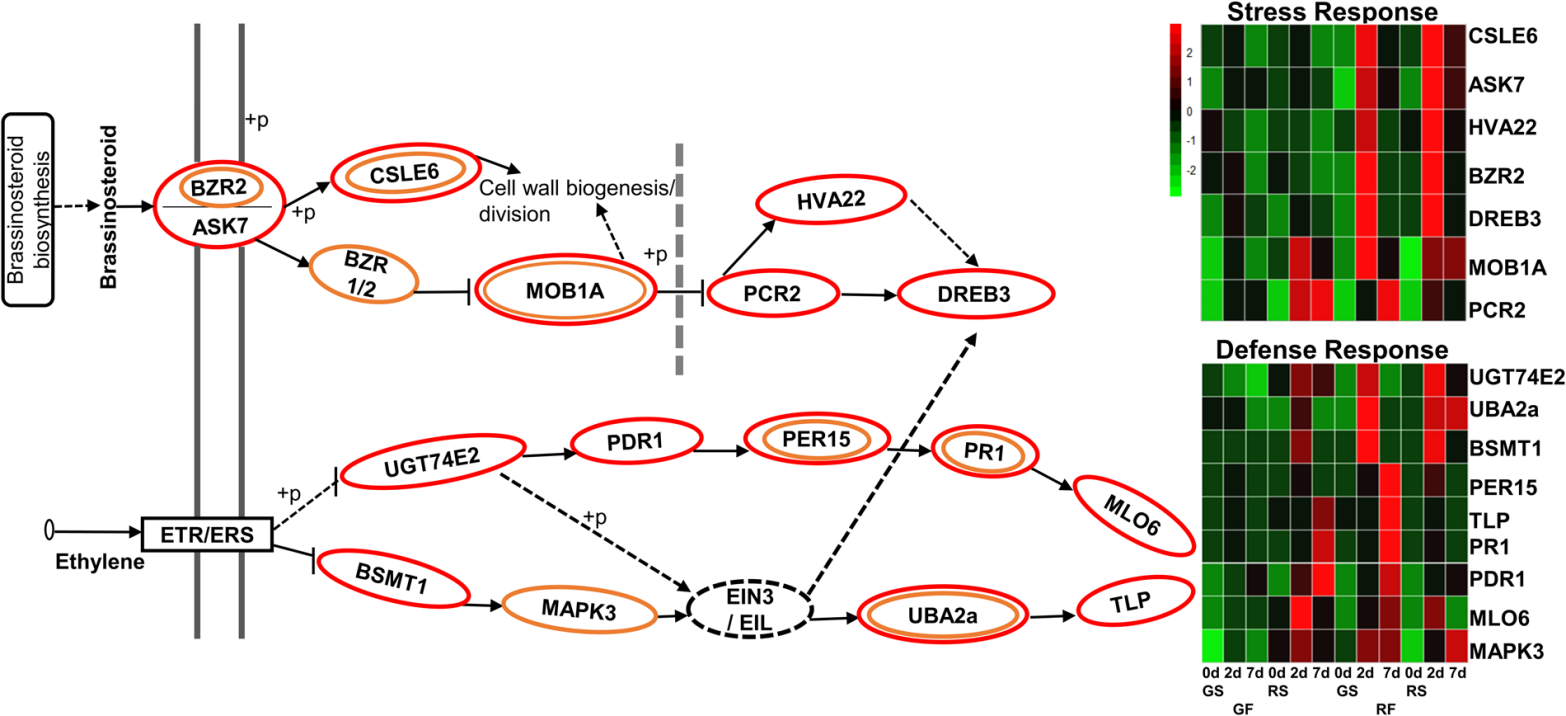
Upregulation of fruit defense and ripening-related pathways in red mango fruit (RF) in response to *C. gloeosporioides* colonization. Fruit defense response related to ethylene, and stress response related to brassinosteroid were upregulated in RF and are presented in signaling pathways and heat maps. Genes circled in red and orange are significantly upregulated on the red side (RS) and green side (GS) of the RF, respectively, in response to *C. gloeosporioides* colonization. *Z*-scores represent rescaled normalized log2 fold-change values. Abbreviations, transcript identification, and expression profile are presented in Table S3A. GF, green fruit

The DEGs in clusters 1, 2, 10, and 11 that were upregulated in the RF in response to *C. gloeosporioides* were mapped to the KEGG database (http://www.genome.jp/kegg/). Several of the upregulated DEGs on both sides of the RF were related to ethylene biosynthesis, which is associated with defense response (Fig. 5). Interestingly, a significant increase of seven transcripts (*ASK7, BZR2*, *CSLE6*, *DREB3, HVA22*, *MOB1A*, *PCR2*) related to brassinosteroid biosynthesis associated with the stress response were upregulated during infection on the red and green side of the RF, along with nine transcripts for ethylene biosynthesis-related to the defense response (*BSMT1, MAPK3, MLO6, PDR1*, *PR1, PER15*, *TLP, UBA2a, and UGT74E2*) (Fig. 5 and Supplementary Table S3).

**Fig. 6:**
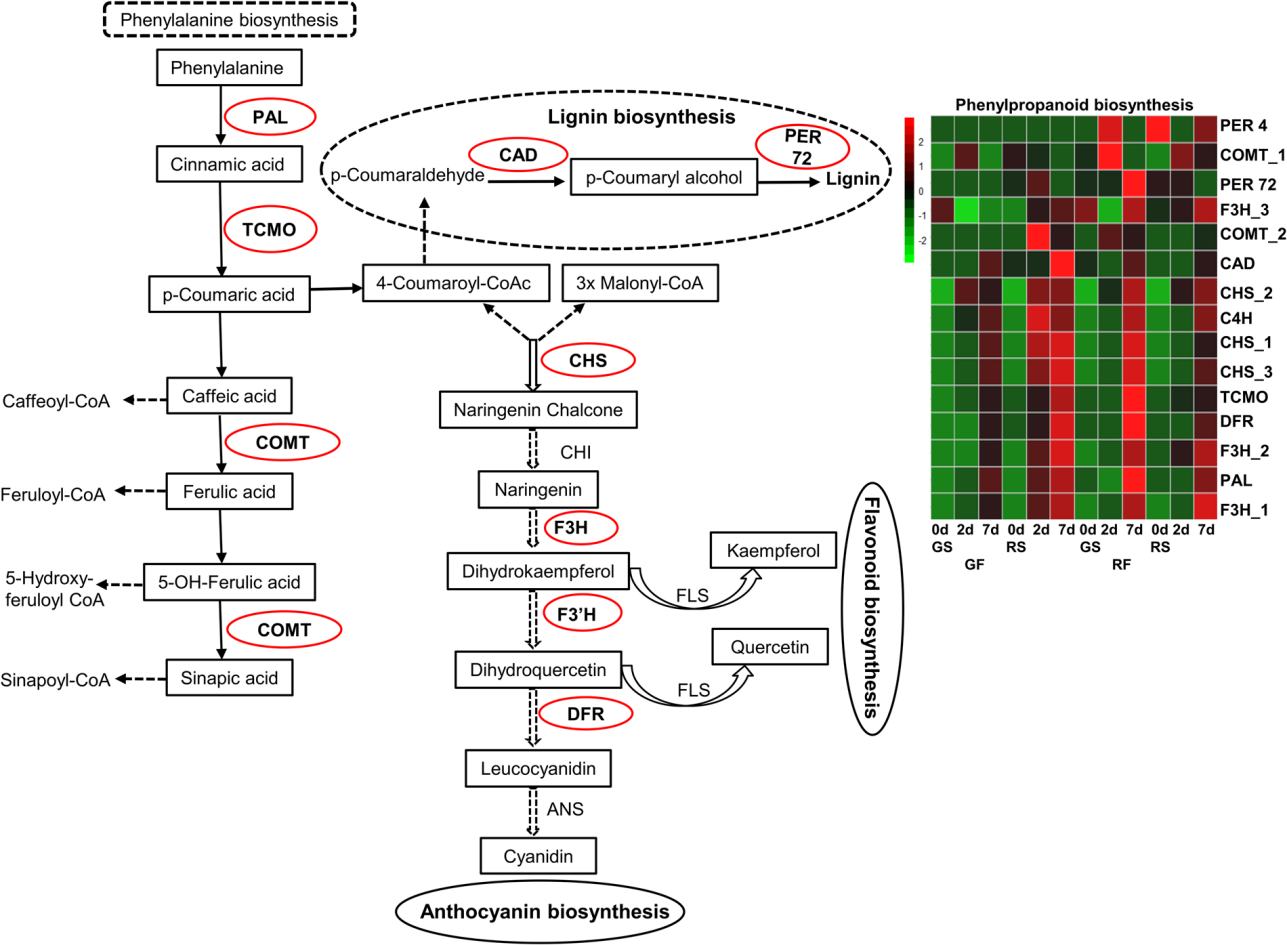
Upregulation of phenylpropanoid-biosynthesis pathway in red mango fruit (RF) in response to *C. gloeosporioides* colonization. Presented are the phenylpropanoid-biosynthesis pathway and the heat map. Genes circled in red are significantly upregulated on the red side (RS) of the RF in response to *C. gloeosporioides*. *Z*-scores represent rescaled and normalized log2 fold-change values. Enzymes in each step: PAL, phenylalanine ammonia-lyase; TCMO, Trans-cinnamate 4-monooxygenase; CAD, cinnamyl alcohol dehydrogenase; PER, Peroxidase; COMT, caffeic acid O-methyltransferase; CHS, chalcone synthase; F3H, flavanone 3-hydroxylase; F3′H, flavanone 3′-hydroxylase; DRF, dihydroflavonol 4-reductase. GS, green side; GF, green fruit

Some of the significant pathways that were induced in RF in response to *C. gloeosporioides* colonization were the phenylalanine-, phenylpropanoid-, lignin-, flavonoid- and anthocyanin-biosynthesis pathways. These connected pathways included 15 DEG transcripts (*CAD, C4H*, *PAL, PER4*, *PER72*, *TCMO*, two transcripts of *COMT*, three transcripts of *CHS*, three transcripts of *F3H*, and *DFR*) (Fig. 6 and Supplementary Table S3).

## Discussion

A number of environmental factors, including high light and UVB radiation, cold temperature, and water stress, can induce the phenylpropanoid pathway and anthocyanin biosynthesis^1,16^. These red-colored mango fruits are more resistant to chilling^7^ and natural decay and probably induce the phenylpropanoid and anthocyanin-biosynthesis pathways^8^. In this study, we found that RF that was exposed to sunlight at the orchard was more resistant to *C. gloeosporioides* infection and showed significantly smaller necrosis and chlorosis diameters than GF that was grown inside the tree canopy. Interestingly, we found that the RF were also more resistant to the fungus on their green side (Fig. 2a, b). However, while the RF were more resistant to *Colletotrichum*, their ripening parameters (similar softening, a slight increase in TSS and decrease in acidity) were similar to those of GF harvested on the same day from the same orchard (Supplementary Fig. S1).

Both anthocyanins and flavonoids have pleiotropic effects and are known to be involved in plant protection against pathogens^1^. Flavonoids and anthocyanins play a significant role as antifungal compounds^9^. In the current manuscript, we show that flavonoids and anthocyanins are present in their glycosylated form in the fruit skin and that their amounts decrease 2- to 3-fold as the fungal infection progresses (Fig. 2). LC–MS characterization showed that the red side of the RF had the highest contents of flavonoids and anthocyanins, compared to their green side and to both sides of the GF (Fig. 1). In mango cv. Kent, there are two major anthocyanins and eight flavonols, detected in the fruit skin by HPLC analysis^4^. Similarly, in the current LC–MS characterization, two anthocyanins and eight flavonols of glycosylated quercetin and kaempferol were detected in the fruit skin of mango cv. Shelly (Fig. 1a, b). This resulted in a high correlation of *R*^2^ = 0.824 between our previous HPLC analysis results with cv. Kent and the current LC–MS results with cv. Shelly.

Both flavonols and anthocyanins are known to have antioxidant activity^7,17^. As expected, infected RF that had 2- to 3-fold more flavonoids showed a 2- to 3-fold increase in antioxidant activity compared to infected GF. Therefore, the RF had lower ROS levels at harvest than the GF. This reduction in ROS was tightly correlated with the higher levels of anthocyanins and flavonols, and high antioxidant activity in the red mango fruit (Fig. 2c–f). Spontaneous photon emission has been shown to reflect lipid oxidation in vegetative plant tissues^14^, and fruit tissue^15^. In this manuscript, GF had a significant (2-fold) increase in luminescence intensity compared to the RF (Supplementary Fig. S3A and B). These results indicate that the fewer flavonols and anthocyanins, lower antioxidant activity, and therefore more ROS in GF lead to higher lipid peroxidation than in RF.

Transcriptional analysis has been widely used to study plant-pathogen interactions and can offer valuable molecular-level information. In addition to innate immunity, in response to pathogen attack, fruit have evolved complex systems of induced resistance^18^. In this study, we found that the red fruits were also resistant on their green side (Fig. 2). To determine whether the induced defense response plays a part in the response to *C. gloeosporioides* on the green side of the RF, we evaluated the upregulated genes in this part of the RF skin at 2 and 7 dpi (Supplementary Fig. 4A, B).

The plant’s defense response to biotic/abiotic stresses is regulated by transcription factors^19^. In this study, 22 transcription factors were upregulated during the host response to *C. gloeosporioides* infection and were significantly increased on the green side of the RF, showing induced resistance (Fig. 4c). Among these transcription factors, *WRKY13* and the dehydration-responsive *DREB3* were upregulated in response to infection on the green side of the RF. Similarly, the induction of several *WRKY* transcription factors in response to *C. gloeosporioides* has been shown in tomato fruit^12^. Interestingly, some of these transcription factors, such as *WRKY* and *DREB*, are known as inducers of biotic and abiotic stress-related genes^20,21^. In addition to the induced transcription factors, the green side of the RF showed induction of some signaling pathways that are well-known for their involvement in induced defense against pathogens: jasmonic acid, ethylene, salicylic acid, abscisic acid, and some MAP kinase pathways (Fig. 4d–h and Supplementary Table S2). While most reports indicate that salicylic acid and jasmonic acid are antagonists^22^, in this study, the salicylic acid- and jasmonic acid-biosynthesis pathways were co-induced (Fig. 4).

To further characterize the red fruit defense response, the clusters of genes that were upregulated in infected red fruit were analyzed. The expression of ethylene-biosynthesis genes such as *ACS* and *ACO* changes during ripening, as previously shown in tomato^23^. Higher levels of ethylene were detected in the green fruit (Supplementary Fig. 2). While, some of the defense-related genes that are associated with ethylene were upregulated during pathogen infection of RF (*BSMT1*, *MLO6*, *MAPK3*, *PR1*, *PDR1*, *PER15*, *TLP, UBA2a*, *UGT74E2*) (Fig. 5). Indeed, ethylene is known to induce plants defense together with jasmonic acid^22^.

Recent evidence indicates that brassinosteroids are also involved in plant-environment interactions and play vital roles in shaping plant fitness and plant growth, as well as in the plant’s innate immunity^24^. Interestingly, one of the more pronounced responses of RF to *C. gloeosporioides* involved brassinosteroid biosynthesis (Fig. 5), indicating its role in the RF’s effective defense response to fungal pathogens.

The phenylpropanoid-biosynthesis pathway is part of the plant’s secondary metabolism, and its branches transform the amino acid phenylalanine into a variety of essential phytochemicals, including lignins, stilbenes, coumarins, salicylates, anthocyanins, and flavonoids^25,26^. As it leads to anthocyanin production, activation of this pathway is also responsible for accumulating red color in mango fruit^4^. Interestingly, mango fruit that developed red skin activated the same pathway of phenylpropanoid biosynthesis in response to fungal attack without reaching anthocyanin biosynthesis (Fig. 6); in this case, the RF upregulated the phenylalanine pathway to produce flavonols and lignin.

To summarize, this study shows that the red mango fruit’s resistance to fungal pathogens is related to the toxicity of flavonols and anthocyanins. However, on the green side of the red fruit, which was not exposed to sunlight in the field and contained less flavonoids, we could also detect activation of the induced defense response. This response included upregulation of several defense-related pathways, among them, jasmonic acid and ethylene, which activated the phenylpropanoid-biosynthesis pathway. Taken together, it seems that fruit exposed to sunlight in the orchard accumulate anthocyanin on the side of the fruit that is exposed to the sun; however, the green side of the RF, which is not directly exposed to the sun, also shows activated fruit defense in response to *Colletotrichum* infection. Thus, exposure to sunlight in the orchard can induce the fruit’s primed and general defense response during postharvest storage.

## Materials and methods

### Plant material

Mango (*Mangifera indica* L., cv. Shelly) fruit were harvested in July 2017. The fruit were picked from two different positions of the canopy in the orchard: the ‘red’ colored fruit (RF) from the exterior position with direct exposure to sunlight, and the ‘green’ colored fruit (GF) from the interior area in the shaded part of the canopy. The fruit was processed within 6 h after harvest and transported from the Mor-Hasharon storage house to the Volcani Center, Israel. Uniform, unblemished fruit weighing approximately 400 g was selected based on skin color: RF with more than 60% red-colored skin and GF with less than 10% red-colored skin. The fruit was washed with tap water and air-dried.

### Evaluation of physiological parameters of skin color for RF and GF

Mango fruit skin color was measured at harvest using a Chroma Meter CR-400/410 (Konica Minolta, Osaka, Japan) at two points on the equatorial line of each fruit (20 measurements per treatment). Ripening and physiological parameters-firmness (Newton), Brix (total soluble solids [TSS]), and total acidity (citric acid equivalence) of RF and GF were measured at harvest according to the method followed by ref. ^7^.

### Fruit inoculation and evaluation of resistance in RF and GF

Freshly harvested ‘Shelly’ mango fruit was disinfected with 1% chlorine for 2 min and rinsed twice with autoclaved water, than wound-inoculated with conidia of *C. gloeosporioides* strain Cg-14^12^. A 7-mL aliquot of conidial suspension (0.5 × 10^6^ conidia mL^−^^1^) was placed into 1-mm deep, 1-mm diameter inoculation spots on the RF and GF pericarp. Four inoculation spots were spaced widthwise on each of 60 fruit per treatment, two inoculations on the green side of the fruit, and two inoculations on the red side of the fruit (120 inoculations per treatment). Decay diameters were measured 2, 4, 7, 8, 10, and 12-day post-inoculation (dpi).

### Ethylene and CO_2_ respiration rates in infected mango fruit

‘Shelly’ mango fruit were inoculated with *C. gloeosporioides* (as mentioned above), and respiration rates of nine red (RF) and green (GF) mango fruits were measured by enclosing each fruit in a 2-L glass jar for 1 h. Gas samples were drawn from the glass jar with a syringe, and the samples were analyzed by gas chromatography for ethylene (Varian-3300, Agilent, Santa Clara, CA, USA) and CO_2_ (GC-2014, Shimadzu, Tokyo, Japan). The ethylene and CO_2_ production rates were measured at 0, 2, 4, 7, and 10 dpi.

### Determination of flavonols and anthocyanins by LC–MS/MS

Skin tissues of RF and GF were extracted by resuspending 0.3 g FW samples in 0.1% (v/v) formic acid in 70% methanol. Samples were filtered through a Millex-HV Durapore (PVDF) membrane (0.45 μm) before injection into the LC-MS instrument. Mass spectral analyses were carried in an ultraperformance LC-quadrupole time-of-flight (UPLC–QTOF) instrument (Waters Premier QTOF, USA), with the UPLC column connected online to a photodiode array detector (Waters Acquity), and then to the MS detector equipped with an electrospray ion (ESI) source (performed in ESI-positive mode). The analytical method is followed by^27^. MassLynx software version 4.1 (Waters Inc.) was used to control the instrument and calculate accurate masses. A mixture of standard compounds was used for instrument quality control. The flavonols and anthocyanins were identified based on standards^4^.

### Total flavonoid and anthocyanin contents, and 2,2-diphenyl-1-picrylhydrazyl (DPPH) and reactive oxygen species (ROS) activity in infected RF and GF

Total anthocyanin content in skin extracts of *C. gloeosporioides*-infected RF and GF at 0–7 dpi was determined by spectrophotometry after organic methanol extraction of the skin followed by absorption measurement at 528 nm^7^. Total flavonoid content in the infected RF and GF skin at 0–7 dpi was extracted and measured using the aluminum chloride colorimetric method^9^; different concentrations of quercetin were used as a standard for quantification. The absorbance was measured at 415 nm in a UV spectrophotometer, and total flavonoid contents were determined in triplicate. Results were expressed as milligram quercetin in 1 g of the sample.

The DPPH radical-scavenging activity of infected RF and GF skin extracts was estimated according to the method followed by^28^ with slight modifications. In this assay, antioxidants in the sample reduce the DPPH radicals, which absorb at 517 nm. A different concentration of ascorbic acid was used as a reference standard for quantification; the reaction was carried out in triplicate.

To determine ROS production in the infected RF and GF, fruit skin (200 µm thickness) was taken from the inoculations zones on the red and green sides of the RF and GF, and incubated with 10 µM 2,7-dichlorodihydrofluorescein diacetate (H_2_DCF-DA) in phosphate-buffered saline (1X PBS) for 15 min in the dark, then washed twice with 1X PBS. The stained skins were observed under a fluorescence microscope (Olympus-BX53, Tokyo, Japan) using GFP3 excitation and emission wavelengths. The relative intensity of the fluorescent signal was calculated using Image J software, as the average intensity from three focal planes in three biological repeats for each sample at different time points (0–7 dpi).

### Evaluation of lipid peroxidation and fluorescence

Infected RF and GF were randomly selected at different time points (0–10 dpi) to detect lipid peroxidation level and natural fluorescence using an in-vivo imaging system (IVIS; PerkinElmer, Waltham, MA, USA). The fruit were kept in the dark for 2 h prior to evaluation. Lipid peroxidation was detected and visualized by auto-luminescence for 20 min with emission at 640–770 nm, as described previously^15^. The fruit’s natural fluorescence intensity was detected by excitation at 495 nm for 2 s and emission at 517–530 nm. The auto-luminescence and fluorescence were recorded with a CCD camera. The optical luminescence and fluorescence image data are presented as the intensity in terms of radiance (photons s^−1^ cm^−2^ steradian^−1^). All measurements were performed in three biological replicates per treatment.

### RNA processing and transcriptome sequencing

A 1-g sample of skin tissue of RF and GF infected with *C. gloeosporioides* was taken from the leading edge of the inoculation area; each treatment consisted of four fruits, two inoculation spots for each side (green and red) of the fruit, and three biological repeats. Total RNA was extracted from the skin tissue as described previously^29^. RNA quality and quantity were determined using an ND1000 UV–VIS spectrophotometer (NanoDrop Technologies, Wilmington, DE, USA). The RNA was treated with DNase and purified (TURBO DNA-free Kit, Ambion Life Technologies, Carlsbad, CA, USA). An RNA integrity number >8.0 was confirmed using a Bioanalyzer 2100 (Agilent Technologies).

The RNA was subjected to deep sequencing by the TranSeq 3′‐end sequencing method for 60 bp single-end reading (Nancy and Stephen Grand Israel National Center, Weizmann Institute of Science, Israel) based on the method described in ref. ^30^. The raw reads were subjected to quality trimming (higher than 15) using the SAMtools suite^31^. All RNA-Seq raw data are available in the NCBI Sequence Read Archive (SRA) under accession number PRJNA575336.

### Data analysis, annotation, and differential expression analysis

The ‘TranSeq’ 3’-end sequencing method for high-throughput transcriptomic analysis was performed according to^30^. The raw reads of 36 libraries were subjected to quality trimming, filtering, and adapter removal by Trimmomatic software^32^. Cleaned sequences were mapped to a reference mango transcriptome^15^ using the TopHat2 software alignment protocol^33^. Bioconductor edgeR^34^ was used to identify differentially expressed transcripts for each biological replicate, based on the count estimates for each transcript. Transcript counts were normalized by calculating reads per kilobase per million (RPKM)^35^, and differentially expressed genes (DEGs) were defined by considering log 2 of fold-change lower than -1 or greater than 1, and false discovery rate (FDR) lower than 0.05.

The genes were annotated^15^ by BLASTX, and assigned a gene ontology (GO) term by combining BLASTX data and InterProScan analysis^36^ using the Blast2GO v2.5 software pipeline^37^. The GO-enrichment analysis was carried out via Fisher’s exact test with multiple testing correction of FDR. Transcripts that were more than fivefold differentially expressed with an FDR-corrected statistical significance smaller than 1e^-5^ were considered differentially expressed. The expression patterns of the transcripts at different time points were studied using cluster analysis of differentially expressed transcripts in at least one pairwise comparison. Expression normalization was calculated using a trimmed mean of *M*-values. Then, hierarchical clustering of transcripts and biological replicates was performed, and hierarchical clusters were extracted based on the Euclidean distance matrix (with the R scripts hclust function). Principal component analysis (PCA) and 2D hierarchical clustering were performed on normalized data using the princomp function in R.

### Transcriptomic analysis of *C. gloeosporioides* in mango fruit

A method similar to that for the mango transcriptome^12^ was followed for *C. gloeosporioides*. The RNA-Seq reads were mapped to the annotated *C. gloeosporioides* genomes with TopHat (*a* = 10, *g* = 5)^38^ and transformed into counts per annotated gene per sample with the ‘coverage Bed’ function from the BED tools suite^39^ and custom R scripts. DEGs between two developmental stages were detected using the ‘exact Test’ function from edgeR^34^. Transcripts with a significant *P-*value (<0.05) and more than two=fold change in transcript level were considered to be DEGs. All *P-*values were corrected for false discoveries resulting from multiple hypothesis testing using the Benjamini–Hochberg procedure.

### qRT-PCR analysis

Single-stranded cDNA was synthesized from 1 μg total RNA from the skin tissue of RF and GF using the Verso cDNA synthesis kit (Thermo Fisher Scientific, Waltham, MA, USA). The synthesized cDNA was used as a template for qRT-PCR analysis to estimate the relative expression levels of the selected genes (*HSP, LOX, UBQ10-1, MAPK16, PAL, F3H*_*-*_*1, and F3H*_*-*_*2*; Supplementary Table S5), followed by the method of ref. ^12^. All samples were normalized using the Ct value of the reference gene (actin; Supplementary Table S5), and values were expressed relative to the control sample.

### Statistical analysis

The data are presented as mean value ± standard error (SE). The physiological data were analyzed by t-test and one-way analysis of variance (ANOVA, Tukey–Kramer HSD test) using JMP (JMP Pro 14 software, SAS Institute, Cary, NC, USA). Different letters indicate significant differences between groups at *p* < 0.05.

## Supplementary information

Supplementary figures (S1-S7)

Supplementary tables S1-S5
