# Supplementary material for: Induced defense response in red mango fruit against Colletotrichum gloeosporioides
Source: Hortic Res. 2021 Jan 10;8:17. doi: 10.1038/s41438-020-00452-4 (PMC7797005; doi:10.1038/s41438-020-00452-4)
Supplement: Supplementary file 1 — Supplementary figures (S1-S7) [file 41438_2020_452_MOESM1_ESM.docx]

**Induced defense response of red mango fruit against *Colletotrichum gloeosporioides***

Pradeep Kumar Sudheeran^1^, Noa Sela^2^*,*Mira CarmeliWeissberg^3^,Rinat Ovadia^4^, ^,^Sayantan Panda^5^, Oleg Feygenberg^1^, Dalia Maurer^1^, Michal Oren Shamir^4^, Asaph Aharoni^5^, Noam Alkan^1^*

### Supplementary Figures


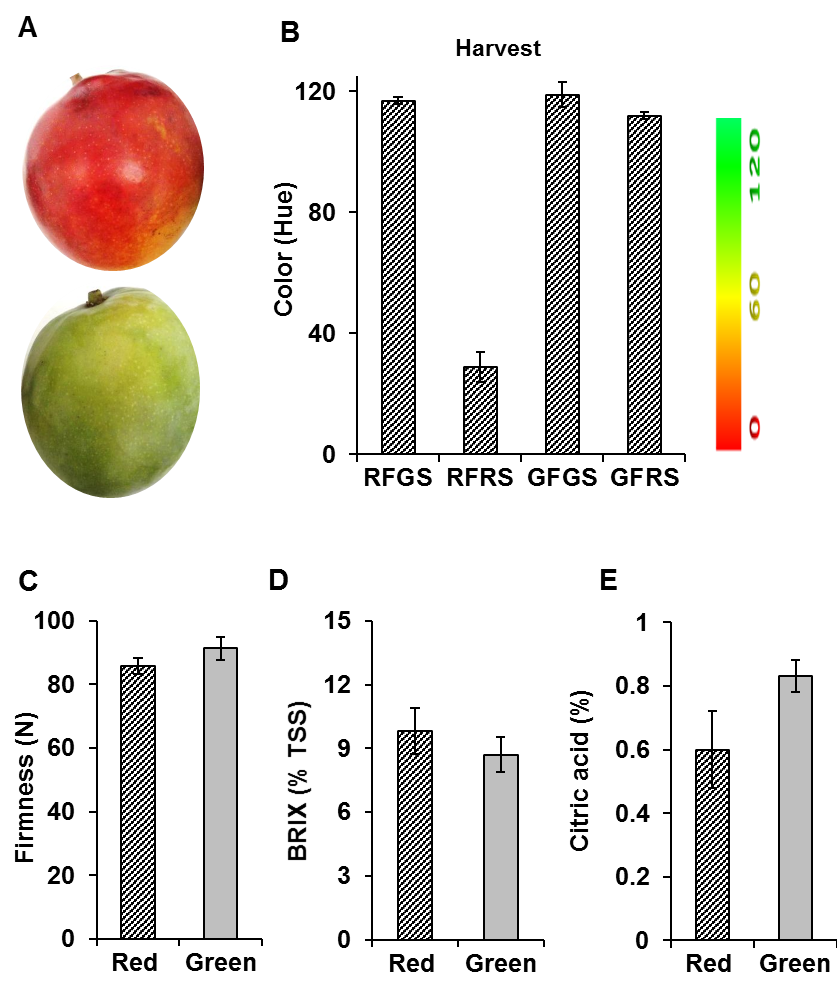


**Fig S1.** Evaluation of red and green mango fruit quality at Harvest. (A). Representative picture of red and green fruit after Harvest. (B). Peel color of red and green fruit at their red and green side, displayed by Hue. Hue color scale bar presented on the right. (C). Fruit firmness in Newton. (D). Brix (total soluble solids; TSS percentage). (E). Acidity (citric acid percentage).


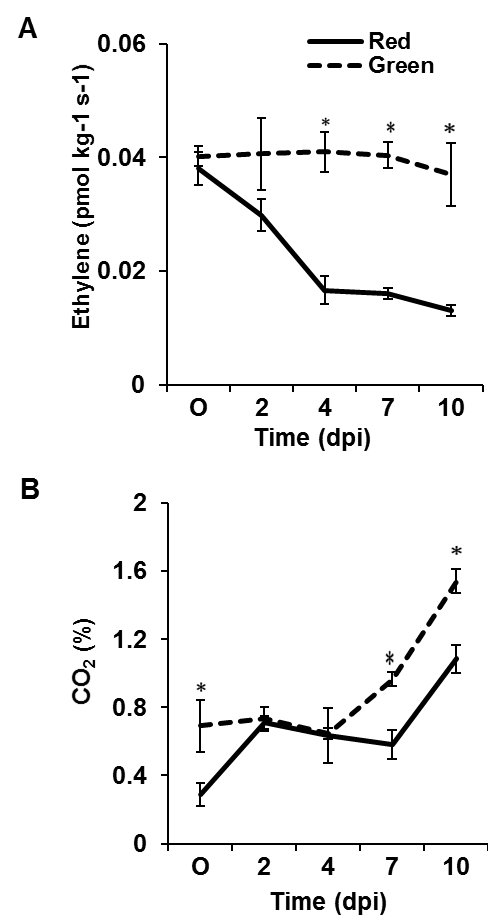


**Fig S2**. Respiration and ethylene in red and green infected mango fruit. (A-B). Ethylene and CO_2_ respiration. Values are means ± SE. Different letters indicate a significant difference (*P*< 0.05) according to Tukey–Kramer HSD tests. Asterix indicates a significant difference (*P*< 0.05), according to the t-test.


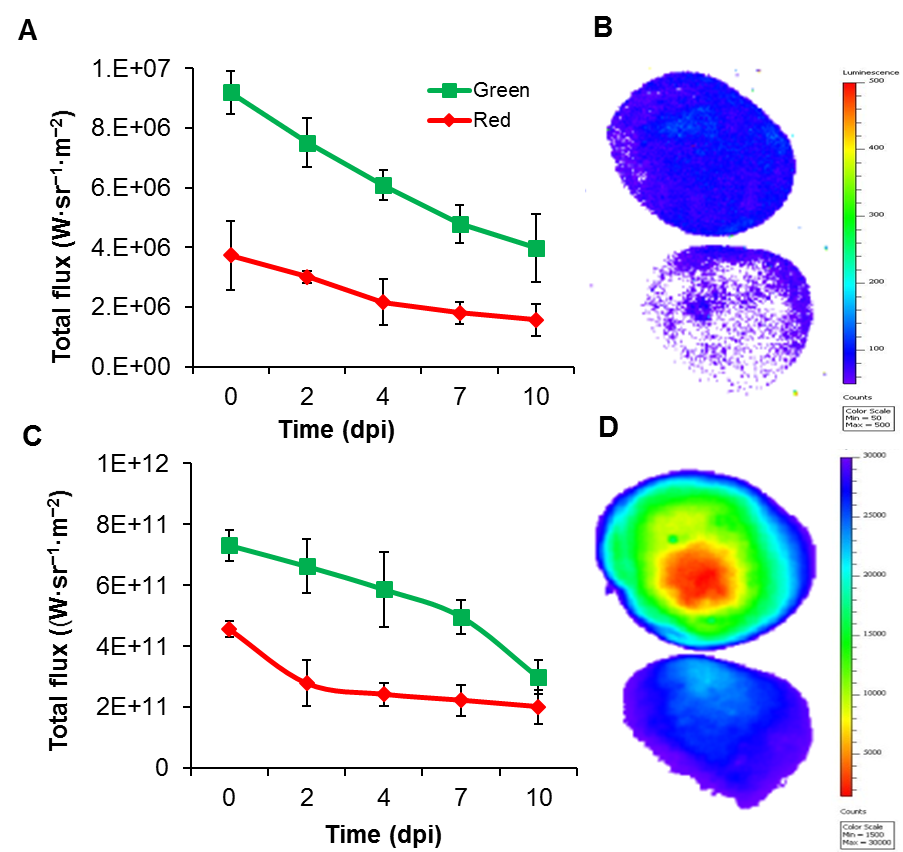


**Fig S3**. Mango fruit fluorescence and luminescence using In Vivo Imaging System (IVIS).(A).Graphical representation of luminescent photon count from red and green mango fruit inoculated by *C. gloeosporioides* for 10 days. (B). Representative picture of luminescence of red and green mango fruit of 7days. (C). Graphical representation of chlorophyll fluorescence intensity. (D). Representative picture of fluorescence of red and green mango fruit of 7 days post-inoculation.


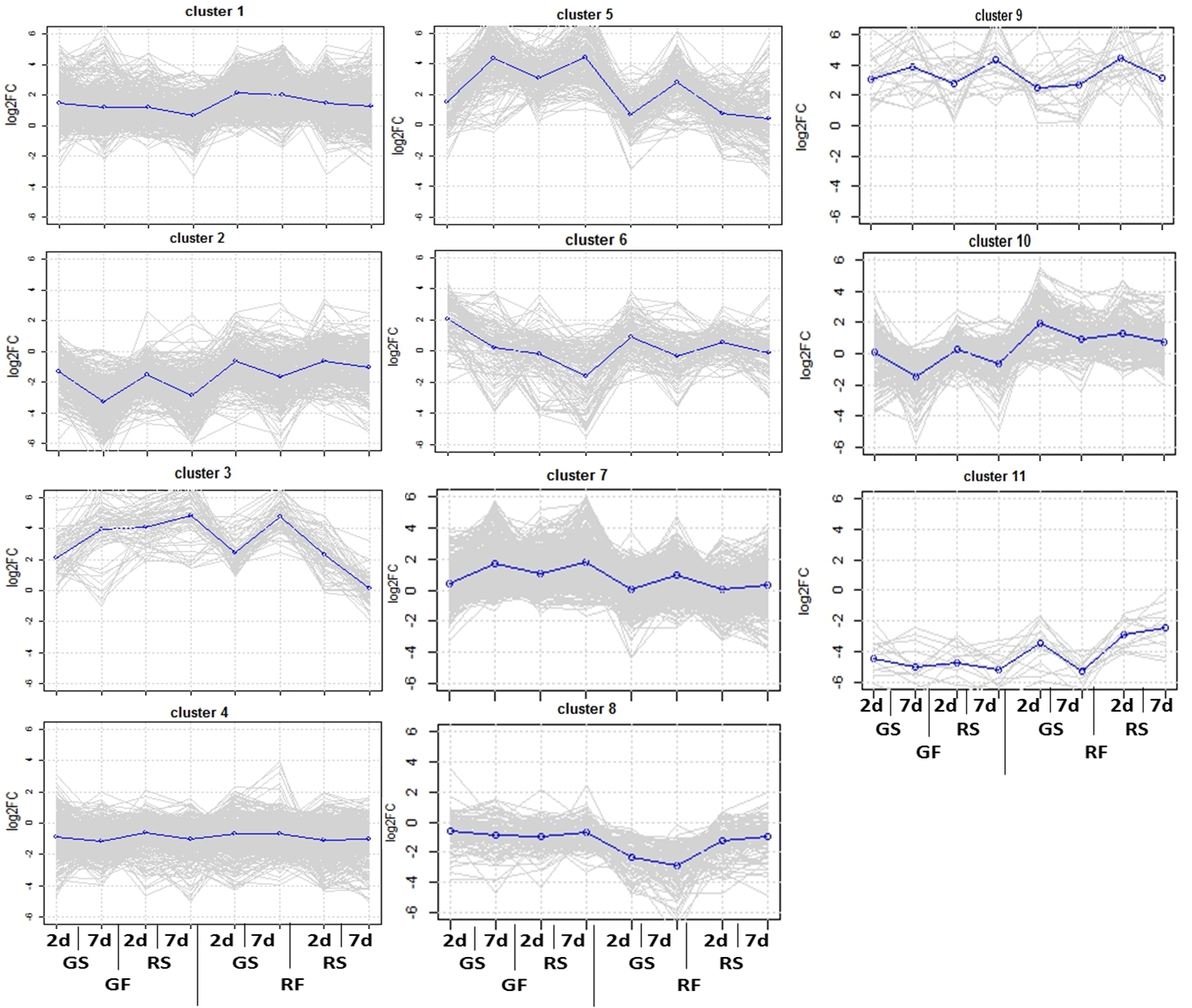


**Fig S4**. Expression patterns of 11 clusters of red and green mango transcriptome in response to *C. gloeosporioides* at different time points. Gray lines mark the various genes profiles, and blue lines represent the average expression profiles in each cluster group. GS, green side; RS, red side; GF, green fruit; RF, red fruit.


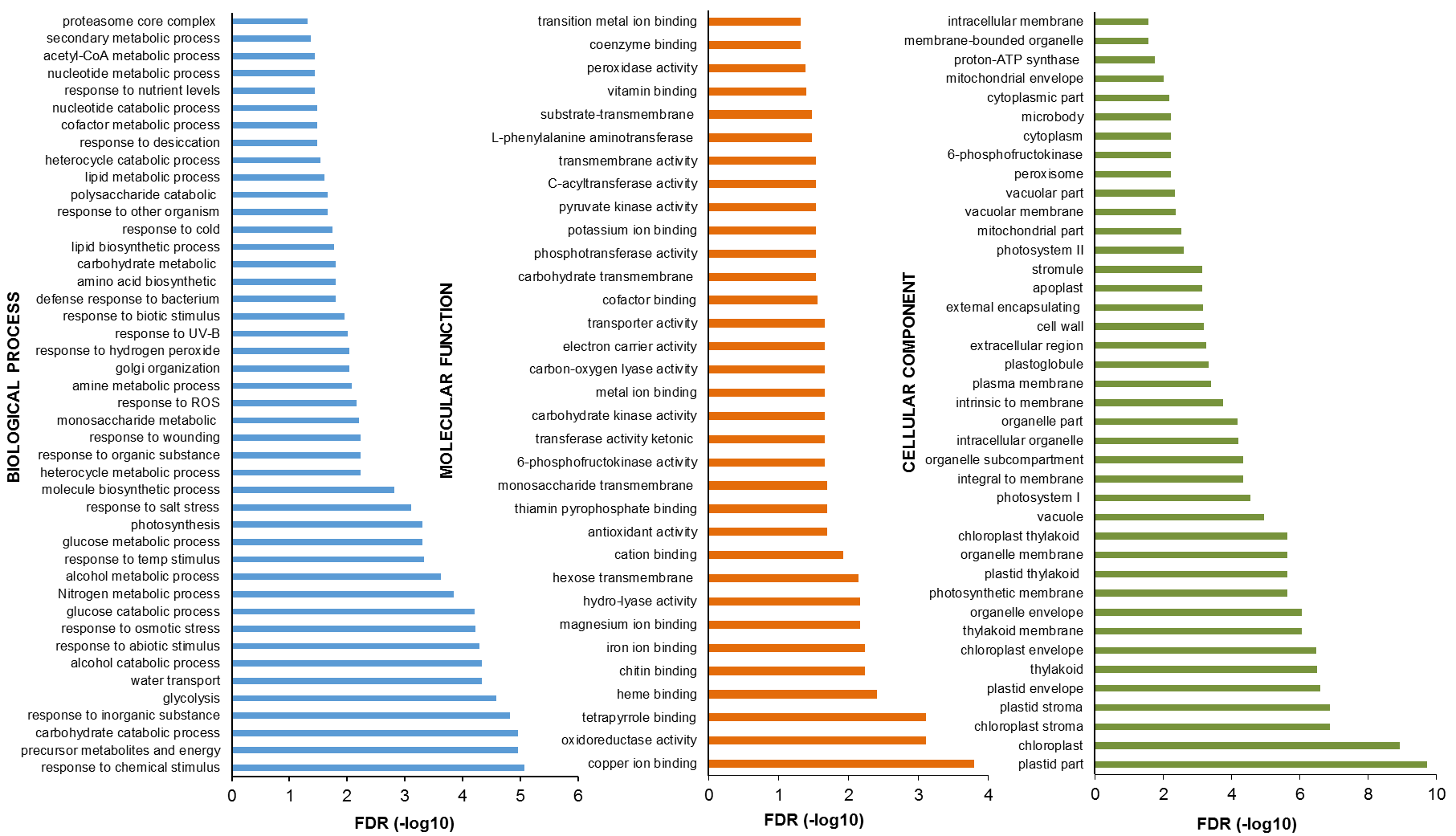


**Fig S5.** Significantly enriched Gene Ontology (GO) of up-regulated transcripts in red fruit, compared to green fruit, in response to *C. gloeosporioides* (clusters 1, 2, 10, and 11)*,* classified into three main categories: biological process, molecular function, and cellular component. The GO term is ranked according to the *p*-value adjusted by FDR (-log10).

**Fig S6.** Correlation between the gene expression obtained from RNA-seq data and qRT-PCR.


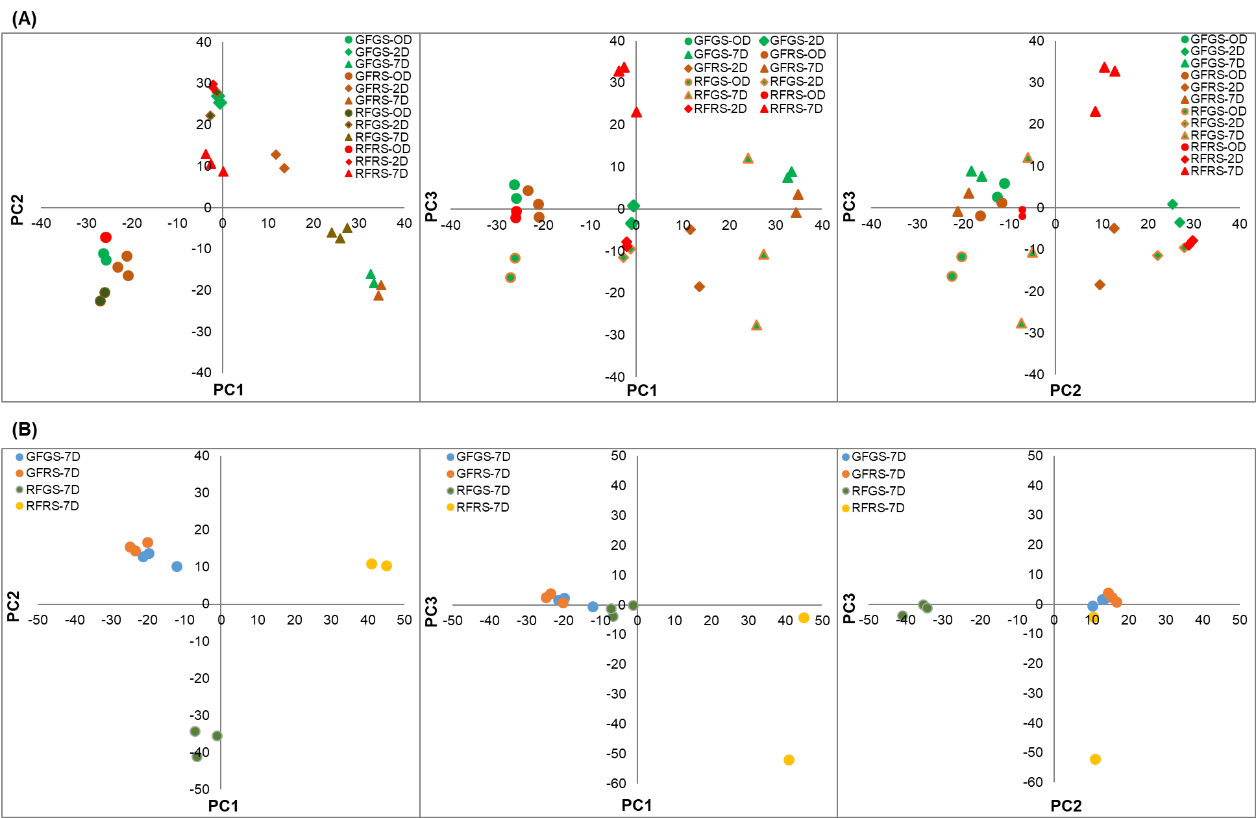


**Fig S7.** (A) 2 dimension PCA of red and green mango transcriptomes on 0, 2, and 7 days post-inoculation. (B) 2 dimension PCA of *C. gloeosporioides* transcriptome on 7D.
